# Treatment with enalapril and not diltiazem ameliorated progression of chronic kidney disease in rats, and normalized renal AT_1_ receptor expression as measured with PET imaging

**DOI:** 10.1371/journal.pone.0177451

**Published:** 2017-05-19

**Authors:** Basma Ismail, Rob A. deKemp, Etienne Croteau, Tayebeh Hadizad, Kevin D. Burns, Rob S. Beanlands, Jean N. DaSilva

**Affiliations:** 1 Cardiac PET Centre, Department of Medicine (Division of Cardiology), University of Ottawa Heart Institute, Ottawa, ON, Canada; 2 Department of Cellular and Molecular Medicine, University of Ottawa, Ottawa, ON, Canada; 3 Kidney Research Centre, Ottawa Hospital Research Institute, University of Ottawa, Ontario, Canada; 4 Department of Radiology, Radio-Oncology and Nuclear Medicine, University of Montreal; University of Montreal Hospital Research Centre (CRCHUM), Montréal, Québec, Canada; University Medical Center Utrecht, NETHERLANDS

## Abstract

ACE inhibitors are considered first line of treatment in patients with many forms of chronic kidney disease (CKD). Other antihypertensives such as calcium channel blockers achieve similar therapeutic effectiveness in attenuating hypertension-related renal damage progression. Our objective was to explore the value of positron emission tomography (PET) imaging of renal AT_1_ receptor (AT_1_R) to guide therapy in the 5/6 subtotal-nephrectomy (Nx) rat model of CKD. Ten weeks after Nx, *Sprague-Dawley* rats were administered 10mg/kg/d enalapril (NxE), 30mg/kg/d diltiazem (NxD) or left untreated (Nx) for an additional 8–10 weeks. Kidney AT_1_R expression was assessed using *in vivo* [^18^F]fluoropyridine-losartan PET and *in vitro* autoradiography. Compared to shams, Nx rats exhibited higher systolic blood pressure that was reduced by both enalapril and diltiazem. At 18–20 weeks, plasma creatinine and albuminuria were significantly increased in Nx, reduced to sham levels in NxE, but enhanced in NxD rats. Enalapril treatment decreased kidney angiotensin II whereas diltiazem induced significant elevations in plasma and kidney levels. Reduced PET renal AT_1_R levels in Nx were normalized by enalapril but not diltiazem, and results were supported by autoradiography. Reduction of renal blood flow in Nx was restored by enalapril, while no difference was observed in myocardial blood flow amongst groups. Enhanced left ventricle mass in Nx was not reversed by enalapril but was augmented with diltiazem. Stroke volume was diminished in untreated Nx compared to shams and restored with both therapies. [^18^F]Fluoropyridine-Losartan PET allowed *in vivo* quantification of kidney AT_1_R changes associated with progression of CKD and with various pharmacotherapies.

## Introduction

Chronic kidney disease (CKD) is a growing health problem worldwide with increasing annual incidence at a rate of 8%, and consumes up to 2% of the global health expenditure [1]. The prevalence of hypertension is substantial in patients suffering from kidney disease [2], and is considered a major risk factor for progression to end-stage renal disease, and for cardiovascular complications [3–6], which represent the leading cause of death in CKD [7, 8]. Most recent guidelines recommend the use of renin angiotensin (Ang) system (RAS) inhibitors in the initial antihypertensive regimen due to class-specific renoprotective mechanisms independent of their blood pressure lowering effect [9–11]. RAS blockade with Ang-converting enzyme inhibitors (ACEIs) or AT_1_ receptor (AT_1_R) blockers (ARBs) have been shown in landmark clinical trials to dramatically attenuate the decline in renal function associated with CKD [12–16]. We have identified AT_1_R as a key molecular imaging target because of its direct involvement with the development and progression of CKD and other aspects of renal pathophysiology; and can eventually assist in prediction and monitoring of therapy.

Nevertheless, alternative antihypertensive drugs given alone or in combination were shown to be successful in reaching optimal blood pressure target and in slowing progression of CKD. In this regard, calcium channel blockers (CCBs) are similarly as efficient as RAS blockers at attenuating hypertension-related renal damage progression, when administered during the non-proteinuric stages of CKD [17–21]. The use of dihydropyridine and nondihydropyridine CCBs has been reported to be safe and effective in management of CKD, provided that a tight control of blood pressure was achieved (reviewed in [22–25]).

Our group has synthesized several positron emission tomography (PET) radioligands derived from the clinically used ARBs for imaging of the AT_1_R in kidney [26–30]. Preliminary PET studies with [^18^F]fluoropyridine (FPyr)-losartan radioligand exhibited high binding selectivity for kidney AT1R over AT2R and rapid metabolism in rats, which supported the potential of this tracer for further renal AT1R evaluation. We have successfully demonstrated an *in vivo* reduction of renal AT_1_R cortical expression in rats with CKD at 8–10 weeks post 5/6 nephrectomy (Nx) using [^18^F]FPyr-losartan with PET imaging [31]. The hypothesis of the present work was that PET imaging of AT_1_Rs would aid in identification of the kidney AT_1_R alterations in association with different actions of the drug treatments used in management of CKD.

## Materials and methods

### Animals

All animal experiments were conducted in accordance with the guidelines of the Canadian Council on Animal Care and with approval of the University of Ottawa Animal Care Committee. Male *Sprague-Dawley* rats (200–250 g; Charles River Laboratories, Montreal, Canada) were housed in pairs on a 12h:12h light:dark cycle and fed standard rat chow and water ad libitum. *Sprague-Dawley* rats were subjected to either sham or Nx surgery in two steps one week apart. Ten weeks after surgery, rats (N = 34) were administered 10mg/kg/d enalapril (NxE) [32, 33], 30mg/kg/d diltiazem (NxD) [34, 35] in drinking water or left untreated (Nx) for an additional 8–10 weeks. Enalapril and diltiazem were obtained from Apotex, Inc. (Ottawa, ON, Canada). Drugs were supplied as tablets that were powdered and dissolved in water and administered to animals in the drinking bottles and doses were calculated according to water consumption for each individual rat. Animals were weighed weekly till the end of study. After sacrifice by decapitation, kidney, heart and left ventricle (LV) weights were obtained.

### 5/6 nephrectomy surgical procedure

**Fig 1:**
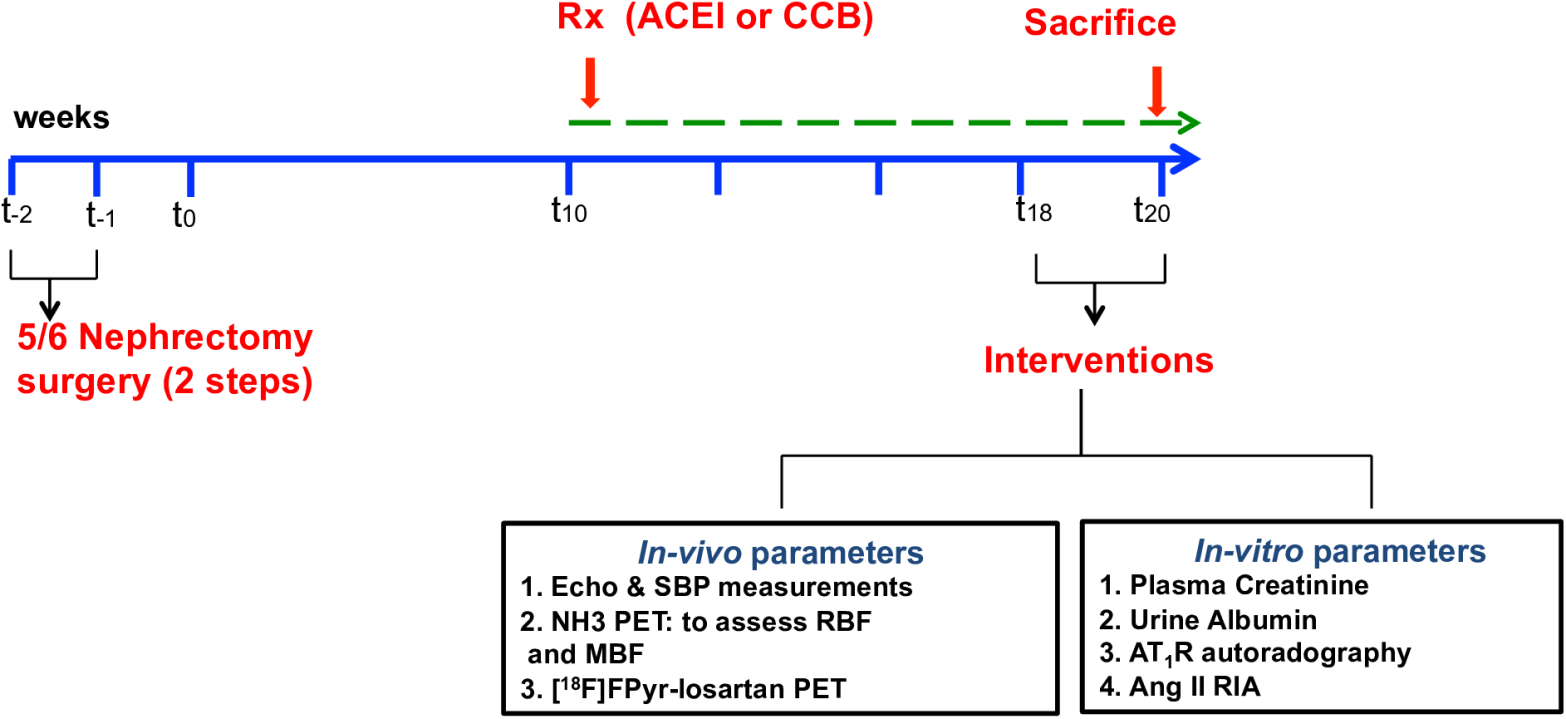
Study timeline of the 5/6 nephrectomy animal model of chronic kidney disease. Treatment (Rx): ACEI (Enalapril 10mg/kg) or CCB (Diltiazem 30mg/kg) started at 10 weeks post-surgery and all parameters assessed at 18–20 weeks post-surgery. SBP: systolic blood pressure; RBF: renal blood flow; MBF: myocardial blood flow; RIA: radioimmunoassay.

Rats were subjected to either sham or Nx surgery in two sittings under total anesthesia with 2% isoflurane by inhalation throughout the surgery. In the first step, the right kidney was exposed through a lateral dorsal incision, then decapsulated and completely removed. One week later, the left kidney was exposed in the same way and reduced to 1/3 of its original size by resecting the superior and inferior poles to induce a total of 5/6 Nx [36, 37]. Post-operative analgesia was provided by subcutaneous administration of buprenorphine twice daily for 3 days following surgery. Sham animals underwent the same 2 surgeries one week apart to simulate Nx conditions without removing the kidneys (Fig 1 shows the study timeline).

### Systolic blood pressure measurements

SBP was measured using indirect tail-cuff plethysmography (CODA-S2 multi-channel, Kent Scientific Corporation, Torrington, CT, USA) in conscious rats at 18–20 weeks’ post-surgery. Animals were placed in rat holders and trained for 3 days to the measuring conditions. On the 4th day, 4–8 consecutive measurements were recorded from each rat per session, and an average blood pressure was calculated.

### Measurement of plasma creatinine

Plasma Cr was assessed at 18–20 weeks post Nx surgery. Blood samples were collected, centrifuged (4000 rpm, 4°C) for 10 minutes, then plasma was obtained and stored at -80°C till assayed. Prior to measuring of Cr in samples, plasma proteins were precipitated using deproteinizing sample preparation kit (Biovision). Cr levels were determined using a Cr assay kit (Cell Biolabs, Inc. San Diego, CA, USA) following the manufacturer’s instructions.

### Measurement of albumin: creatinine ratio in urine

Urinary albumin excretion was determined using albumin:Cr ratio from spot urine collection from rats. Albumin was quantified with a commercially available rat ELISA kit (Genway Biotech, Inc. San Diego, CA, USA) and Cr was measured with same assay kit mentioned in previous section.

### *In vivo* PET imaging

MicroPET studies were performed using the Inveon DPET (Siemens Preclinical Imaging, Knoxville, TN) small animal scanner. Animals were anaesthetized and kept unconscious throughout the scan using 2% isoflurane. Rats were placed on the microPET scanner bed in supine position and body temperature was maintained at 37°C. Animals were positioned to include heart and both kidneys in the field of view (FOV). Body temperature, heart rate and respiratory rate were monitored throughout the scans. A ^57^Co transmission scan was obtained for scatter and attenuation correction either immediately preceding or following the emission scan. Images were analyzed with Inveon® Research Workplace software version 1.4 (Siemens Preclinical Imaging, Knoxville, TN) unless indicated otherwise. Dynamic PET images were reconstructed using vendor-provided 3-dimensional ordered subset expectation maximization / maximum a posteriori algorithm OSEM3D/MAP (β = 1, OSEM3D iterations = 2, MAP iterations = 18) with all corrections enabled. Volumes of Interest (VOIs) were defined on reconstructed images to obtain time-activity-curves (TACs) in units of Bq/cc.

### [^18^F]FPyr-Losartan AT_1_R

[^18^F]FPyr-Losartan was synthesized as previously described and dissolved in 0.9% saline for injection [26, 38]. Rats were injected with 18–81 MBq (0.5–2.2 mCi) of [^18^F]FPyr-losartan (<1ml volume) via a 26 gauge catheter into the lateral tail vein. The specific activity ranged from 112–1630.52 mCi/μmol (4.144–60.33 GBq/μmol) at time of injection. A dynamic 60 min scan was acquired as 12x10 s, 3x60 s, 11x300 s frames. The arterial input function was obtained from the average blood pool activities within the left atrium (LA) or the left ventricle (LV) cavity. Briefly, a sphere was drawn inside the cavity of LA or LV at an early frame (10–40 s post-injection) and an 80% threshold was used to define the contour of the arterial blood VOI. Kidney VOIs were drawn at frame 16 or 17 (5–15 min post-injection where the highest tissue-to-background contrast was observed) by tracing a segment of the cortex at the inferolateral side of the left kidney away from liver and bowel to avoid spillover, and the final VOI was defined using a 50% threshold contour. [^18^F]FPyr-Losartan retention was measured using the Logan derived distribution volume (DV) values. The tracer DV provides a quantifiable parameter for repeated measurements and assessment of repeatability and reliability. Provided the tracer binds to its receptor reversibly, Logan graphical analysis [39], of the PET time-activity data can be used to calculate the DV. In essence, tracer uptake in the kidney is plotted against concentration in the plasma at equilibrium (steady-state). Plotting 0∫TCPET(t)dt/CPET(T)(min) against 0∫TCP(t)dt/CPET(T)(min), where CPET(T) is the concentration of tracer in the tissue and CP(t) is the concentration of tracer in the plasma, will transform the tissue activity to a linear plot, as if the tracer was injected as a continuous infusion. The slope of this line during the steady-state phase corresponds to an estimate of the DV (ml/cm^3^).

### [^13^N]Ammonia blood flow

Heart and kidneys perfusion were assessed within the same week of [^18^F]FPyr-losartan PET scans. Animals were injected [^13^N]ammonia 55–110 MBq (1.5-3mCi) intravenously and scanned for 30 min. Myocardial blood flow (MBF) was quantified using FlowQuant software [40]. Blood and kidney TACs were generated and flow values (ml/g/min) were produced. For calculation of renal blood flow (RBF), the initial 2 min of the dynamic PET data was used to avoid contamination by plasma metabolites of N-13 radioactivity. The images were reconstructed into 12 x 10 s frames applying the corrections for dead-time, isotope decay, detector efficiencies, and random events. Renal TACs were derived from VOI drawn over renal cortex and derived from blood pools inside the LA and LV for average arterial input function. The one-tissue-compartment kinetic model was used to calculate K_1_ (ml/g/min) for RBF analysis.

### *In vitro* autoradiography

*In vitro* ^125^I-[Sar^1^, Ile^8^]Ang II binding was assessed using the method published previously [41]. [^125^I]-Sar^1^, Ile^8^]Ang II was purchased from Perkin Elmer (USA). The reagent was supplied as a powder that was dissolved in distilled water, each vial contained 50μCi (1.85MBq) with a specific activity of 2200Ci (81.4TBq)/mmol. Briefly, rats were sacrificed 2–3 days after PET imaging studies. Following decapitation, dissected kidneys were quickly immersed in OCT Compound (Tissue-Tek), frozen on dry ice and stored at -80°C until subsequent autoradiography studies. Kidneys were sectioned in the longtudinal axis into 20 μm-thick slices at -18°C with a cryostat (Leica CM3050 S). Tissue sections were thaw-mounted on glass slides (VWR) and stored at -80°C. On the day of the experiment, slides were pre-washed in assay buffer (150 mM NaCl, 50 mM sodium phosphate dibasic, 1 mM EDTA, 0.1 mM Bacitracin, 0.1% BSA) for 15 min then incubated with 0.8 nM ^125^I-[Sar^1^, Ile^8^]Ang II for 90 min at room temperature in the presence of AT_2_R antagonist, PD 123,319 (10μM) to determine total (non-AT_2_R) binding or with unlabelled angiotensin II for non-specific binding. Specific binding of ^125^I-[Sar^1^, Ile^8^]Ang II was calculated as total (non-AT_2_R) minus non-specific binding. After incubation, slides were washed 3 times (15 min at 4°C) sequentially in assay buffer, deionized water, and again in assay buffer then gently air dried. Sections were then exposed to phosphor imaging plates (Kodak Screen-K, Biorad) for 48hours in complete darkness. Phosphor plates were then read at a 100 μm resolution (BioRad Molecular Imager FX) and analyzed using Quantity One Software (BioRad, Philadelphia). Quantification was done by manually tracing the whole kidney cortex and the radioactivity density (counts/mm^2^) was recorded for that area.

### Measurements of angiotensin II plasma and tissue levels

Ang II analyses were performed by the Hypertension Core Laboratory at Wake Forest University Health Science Center using previously described method [42]. After decapitation of animals, trunk blood was collected in EDTA tubes containing a cocktail of protease inhibitors including 0.44 mM 1,20 ortho-phenanthroline monohydrate (Sigma, St. Louis MO.), 0.12 mM pepstatin (Peninsula Labs, Belmont CA), and 1 mM Na p-hydroxymercuribenzoate (Sigma, St. Louis MO), then centrifuged at 4000 rpm for 5 min to obtain plasma. Kidneys were rapidly collected and snap frozen on dry ice. Plasma and tissue samples were stored at -80°C until shipped for assay. Tissues were homogenized in an acidic ethanol [80% vol/vol 0.1N HCl] solution containing peptidase inhibitors described previously [43]. Samples were Sep-Pak extracted and measured by RIA (ALPCo, Windham, NH, USA). An aliquot for protein determination was taken from the acid ethanol homogenized tissue. Protein was measured in this aliquot and results are expressed per mg protein.

### Echocardiography

Echocardiography was carried out at 18–20 weeks under light anesthesia (1–2% isoflurane) using the Vevo 770 system (VisualSonics, Toronto, ON, Canada) and a 23.5 MHz probe. All echocardiography studies were performed and analyzed by a single not-blinded operator. Parasternal long-axis views were recorded as sequential ECG-gated M-mode sweeps (EKV-mode) to generate two-dimensional cines of the left ventricle. Analysis of results was completed with the VisualSonics cardiac measurements program. The LV wall mass and LV ejection fraction (LVEF), end diastolic volume (EDV) and stroke volume (SV) were assessed with the standard VisualSonics cardiac measurement formulas.

### Statistical analysis

All data are presented as mean ± standard deviation. Statistical analysis was performed with one way ANOVA using Tukey test for posthoc analysis (Prism; GraphPad Software; version 6.0h); *p*<0.05 was considered significant. N values for each comparison are given in the Figures and Tables. Data are expressed either as mean and standard deviation in the Tables, or medians and interquartiles in the boxplot Figures. Pearson correlation coefficient (r) was calculated to determine correlations between various parameters when required.

## Results

### Body and organ weights

**Fig 2:**
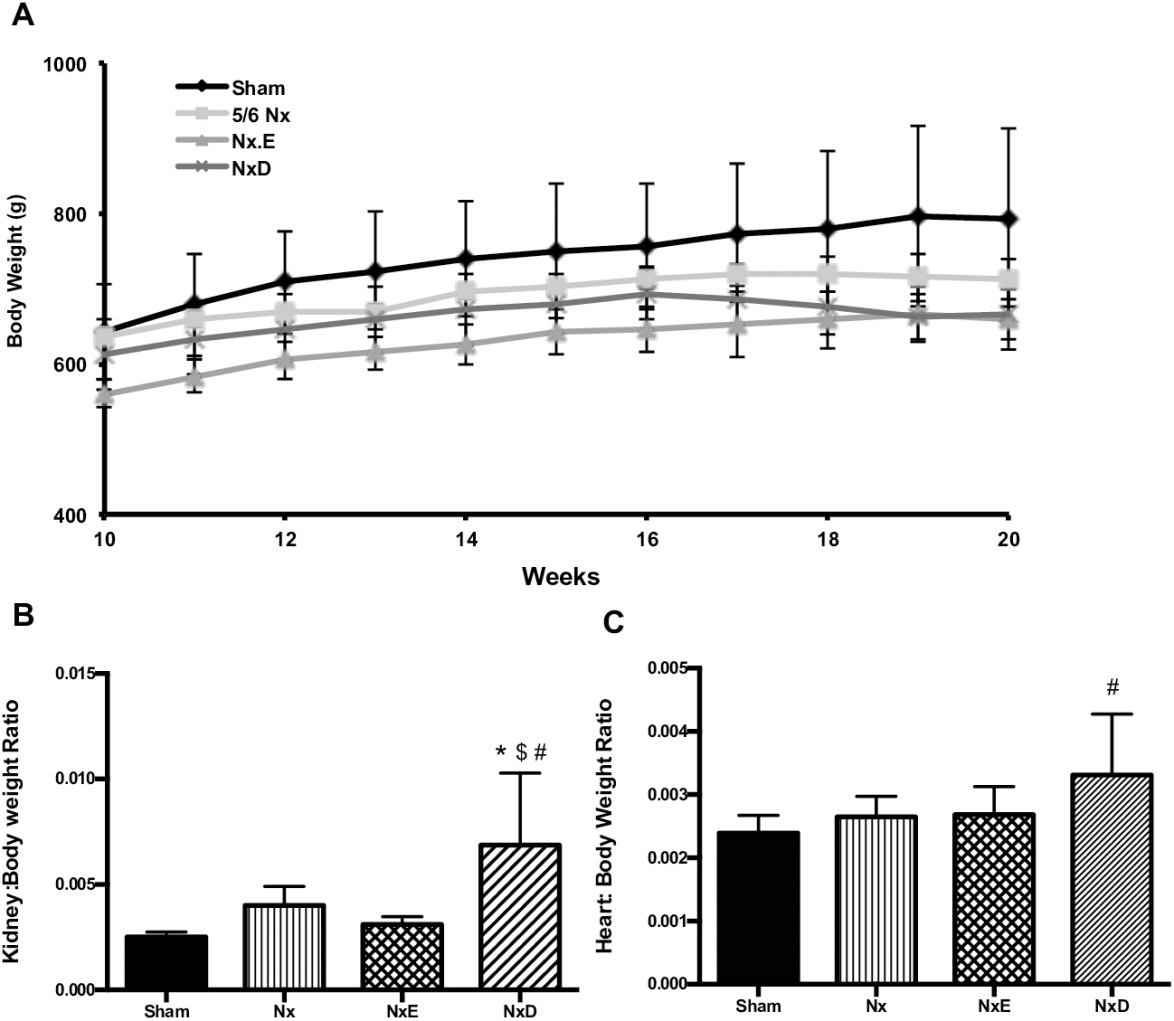
**Body weight data obtained weekly over 10 weeks (A), terminal left kidney weight for sham (N = 8), Nx (N = 8), NxE (N = 6) and NxD (N = 8) (B) and heart weight for sham (N = 8), Nx (N = 9), NxE (N = 9) and NxD (N = 8) (C) normalized to body weight at 18–20 weeks post-surgery.** Data are presented as mean±SD. * *p*<0.05 vs sham, ^$^ *p*<0.05 vs Nx, ^#^ *p*<0.05 vs NxE (p<0.05).

At 8–10 weeks before the start of treatments, body weights were comparable among all groups of animals. Similarly, at 18–20 weeks post Nx, no significant differences in body weight or the percentage increase per week were observed between the groups (Fig 2A). No significant changes in left kidney weight/body weight were observed in Nx or NxE rats, compared to shams, although there was a tendency for kidney hypertrophy in the Nx group. Left kidney weight/body weight was significantly increased in diltiazem-treated rats, compared to all groups (1.7–2.7-fold, *p*<0.05; Fig 2B). Cardiac hypertrophy was not observed in the sham, Nx and NxE groups. However, treatment with diltiazem induced a significant increase in heart weight/body weight compared to shams (1.4-fold, *p*<0.05; Fig 2C).

### Systolic blood pressure and renal function

Nx rats were hypertensive and had significantly higher plasma Cr levels compared to sham rat group before the start of therapy at 8–10 weeks post-surgery as we previously reported [31]. Nx rats had significantly higher SBP at 18–20 weeks post-surgery compared to shams (1.5-fold; *p*<0.001). Both enalapril and diltiazem treatments attenuated this increase (Table 1).

**Table 1:** SBP, plasma creatinine (Cr) and urinary albumin:Cr in sham, untreated Nx, NxE and NxD (at 18–20 weeks post-surgery).

Parameters	SBP (mmHg)	Plasma Cr (mg/dl)	Urine Albumin:Cr (ug/mg)
**Sham**	142.7±12* N = 8	0.48±0.06 N = 5	81.4±30.0 N = 4
**Nx**	188.8±17.3 N = 4	0.77±0.07* N = 6	211.37±39* N = 4
**NxE**	152±8.65^$^ N = 6	0.43±0.06^$^ N = 5	116.5±32.1^$^ N = 4
**NxD**	145.8±5.78^$^ N = 5	1.46±0.27*^$^^#^ N = 6	385.6±53.5*^$^^#^ N = 4

Data are presented as mean±SD.

* *p*<0.05 vs sham

^$^ *p*<0.05 vs Nx

^#^ *p*<0.05 vs NxE (*p*<0.05).

Plasma Cr concentration was significantly higher in Nx rats in comparison to shams at 18–20 weeks post-surgery (1.6 fold; *p*<0.05). By contrast, plasma Cr was not increased in the enalapril-treated group, compared to untreated Nx animals, while diltiazem induced a significant increase compared to all groups (1.8–3 fold; *p*<0.05) (Table 1). At the end of study, significant albuminuria (corrected to urine Cr concentration) developed in the untreated Nx animals, which was normalized by enalapril and exacerbated in diltiazem-treated rats (NxD: 4.8-fold increase versus sham, 1.8-fold increase versus Nx; *p*<0.001) (Table 1).

### Plasma and tissue angiotensin II levels

**Fig 3:**
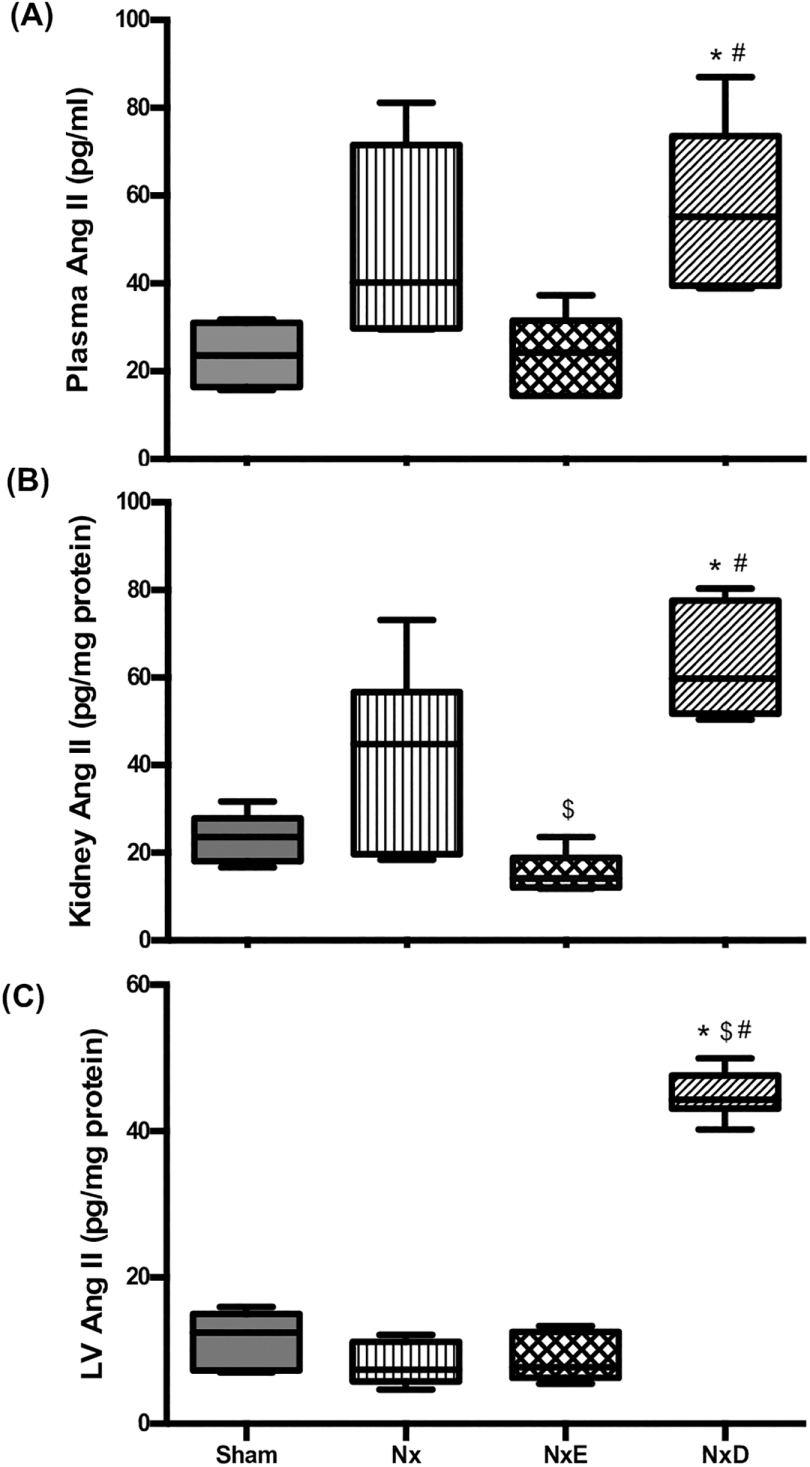
**Ang II levels in plasma samples of sham (N = 4), Nx (N = 5), NxE (N = 5) and NxD (N = 6) (A), left kidney samples of sham (N = 5), Nx (N = 6), NxE (N = 5) and NxD (N = 5) (B) and left ventricle samples of sham (N = 6), Nx (N = 7), NxE (N = 5) and NxD (N = 6) (C) samples of Sham, Nx, NxE and NxD groups at 18–20 weeks post-surgery.** In boxplots, horizontal lines represent median and whiskers represent minimum and maximum values. Data are presented as mean±SD. * *p*<0.05 vs sham, ^$^ *p*<0.05 vs Nx, ^#^ *p*<0.05 vs NxE (p<0.05).

Ang II levels in plasma, kidney and LV were elevated in Nx rats compared to sham, however did not reach significance (*p* value = 0.15, 0.13, 0.22 respectively). Administration of enalapril did not affect Ang II levels in plasma or LV but significantly reduced levels in the kidney compared to Nx rats (*p*<0.05; Fig 3). Interestingly, treatment with diltiazem induced significant elevations in plasma and kidney Ang II levels, in comparison to the sham and NxE groups at 18–20 weeks (*p*<0.05 for plasma and *p*<0.001 for kidney; Fig 3A & 3B). Moreover diltiazem caused a marked elevation of Ang II in LV compared to all other groups (3.75-fold versus sham, 5.6-fold versus Nx, and 5-fold versus NxE; Fig 3C).

### AT_1_R expression

**Fig 4:**
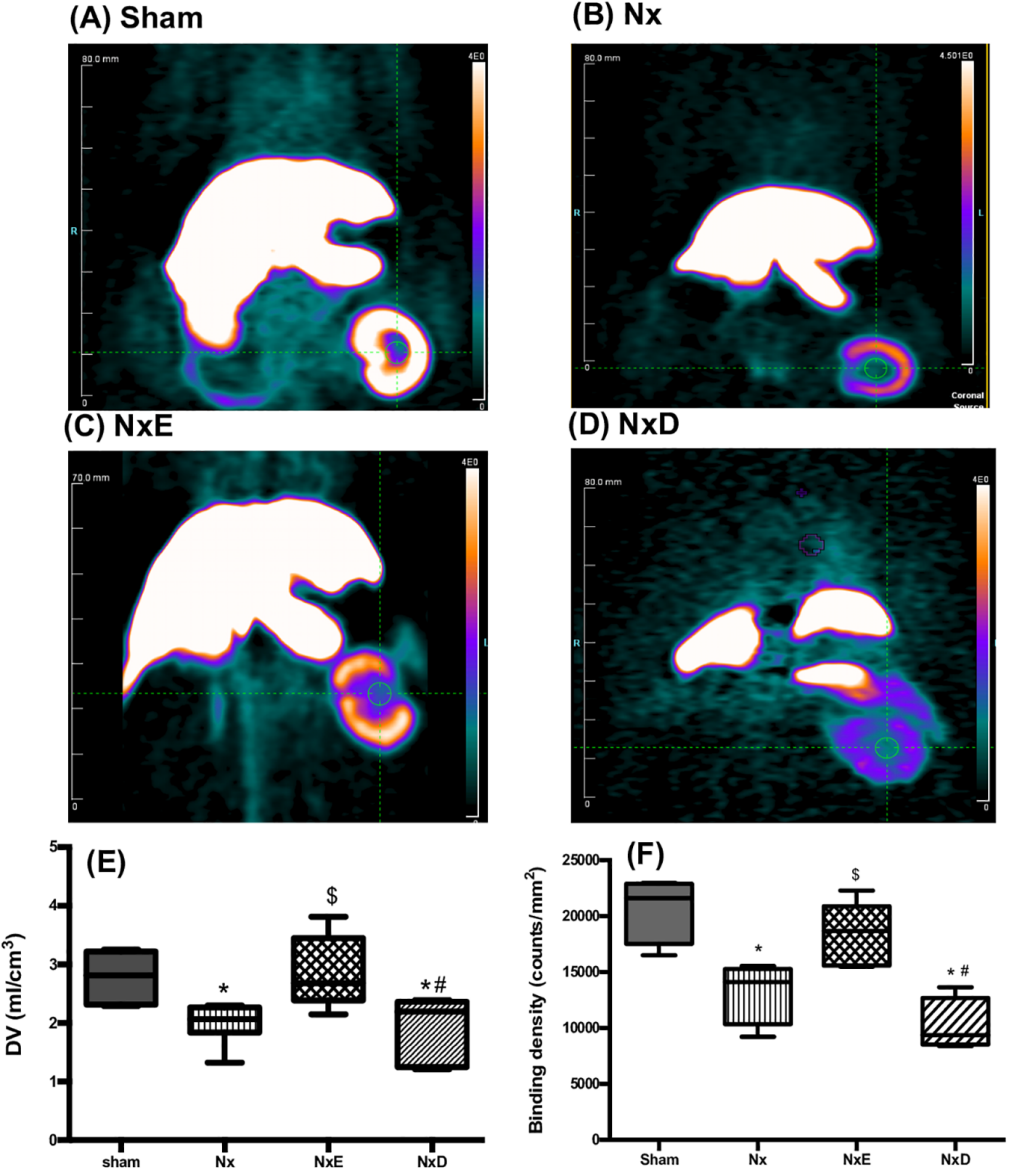
**Representative coronal view microPET scans showing liver and kidney uptake obtained at 5–10 min post-injection of [**^**18**^**F]FPyr-losartan in all groups at 18–20 weeks post-surgery; Sham (A), Nx (B), NxE (C) and NxD (D). Images are displayed using same SUV scale. Kidney distribution volume (DV, ml/cm**^**3**^**) of [**^**18**^**F]FPyr-losartan obtained with PET *in vivo* in sham (N = 6), Nx (N = 7), NxE (N = 7) and NxD (N = 6) (E); and** ^**125**^**I-[Sar**^**1**^**, Ile**^**8**^**]Ang II binding density (counts/mm**^**2**^**) obtained with *in vitro* autoradiography in sham (N = 4), Nx (N = 4), NxE (N = 6) and NxD (N = 4) (F) at 18–20 weeks post-surgery.** In boxplots, horizontal lines represent median and whiskers represent minimum and maximum values. Data are presented as mean±SD. * *p*<0.05 vs sham, ^$^ *p*<0.05 vs Nx, ^#^ *p*<0.05 vs NxE (p<0.05).

*In vivo* PET scans of the Nx rats revealed a distorted shape of the kidney after resection with less tracer uptake as compared to the normal kidney in shams (Fig 4A & 4B). While enalapril preserved the shape of the remnant left kidney and tracer retention, diltiazem-treated rats displayed further distortion of the kidney structure with minimal uptake of the tracer (Fig 4C & 4D). [^18^F]FPyr-Losartan distribution volume (DV, ml/cm^3^) determined by Logan analysis was quantified as an indication of AT_1_R expression in the kidney of rats (Fig 4E). DV values were significantly lower in Nx rats compared to sham animals (-28%, *p*<0.05). Treatment with enalapril normalized these values while no change was observed in the diltiazem-treated group. [^125^I]-[Sar^1^, Ile^8^]Ang II specific binding density confirmed reduction of AT_1_R in the untreated Nx group at 18–20 weeks (-36%, *p*<0.05), and normalization of the receptor expression in rats receiving enalapril but not with diltiazem therapy (Fig 4F). These results correlated well with the *in vivo* PET findings (r = 0.47, *p* = 0.05).

### Organ blood flow

**Fig 5:**
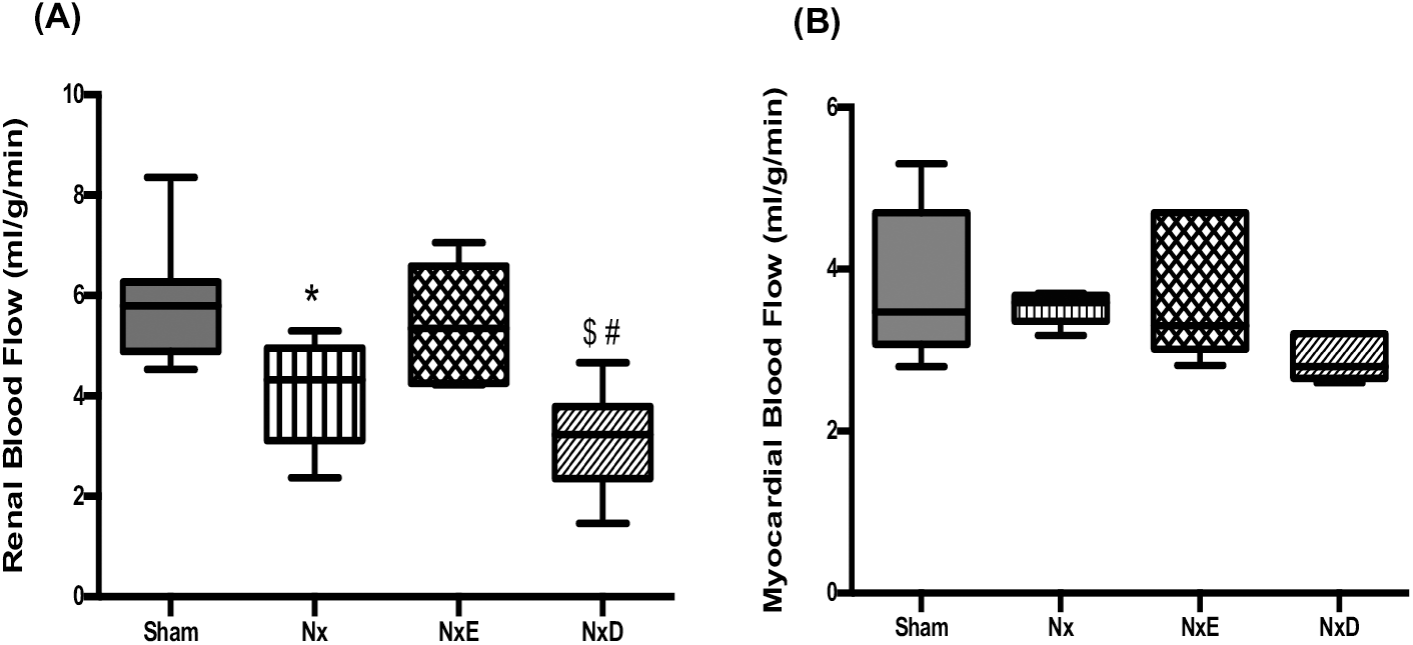
**Renal blood flow in sham (N = 7), Nx (N = 7), NxE (N = 5) and NxD (N = 7) groups (A); and myocardial blood flow values in sham (N = 6), Nx (N = 5), NxE (N = 6) and NxD (N = 5) (B) at 18–20 weeks post-surgery. Blood flow was assessed by [**^**13**^**N]ammonia PET imaging.** In boxplots, horizontal lines represent median and whiskers represent minimum and maximum values. Data are presented as mean±SD. * *p*<0.05 vs sham, ^$^ *p*<0.05 vs Nx, ^#^ *p*<0.05 vs NxE (p<0.05).

Renal blood flow as assessed by [^13^N]ammonia PET was significantly reduced in Nx rats, compared to shams (*p*<0.05), and tended to normalize with enalapril treatment, although this did not achieve statistical significance. By contrast, diltiazem caused a further significant reduction in renal blood flow, compared to untreated Nx rats (Fig 5A). No significant differences in myocardial blood flow occurred amongst groups (Fig 5B).

### Heart function

Echocardiographic measures of heart functions are summarized in Table 2. There was no difference in LVEF between all groups. However, the EDV and SV were significantly diminished in untreated Nx rats as compared to shams. Treatment with enalapril and diltiazem restored only SV to normal levels (Table 2). Echocardiographic LV mass was significantly increased in the Nx group and was not reversed in rats receiving enalapril. Diltiazem treatment further enhanced the LV mass compared to all groups (3.5-fold versus sham, 1.7-fold versus Nx, and 1.7-fold versus NxE; *p*<0.001).

**Table 2:** Echocardiographic parameters in sham, untreated Nx, NxE and NxD groups at 18–20 weeks post-surgery.

Parameters	LV EF (%)	SV (ml)	EDV (ml)	LV mass (g)
**Sham**	73.3±5.3 N = 6	450±89 N = 6	611±91.8 N = 6	449.8±89.03 N = 6
**Nx**	63.6±7.9 N = 5	285±42.3* N = 5	462±74.3* N = 6	923.4±165.32* N = 6
**NxE**	73.3±6.5 N = 9	414±89.4^$^ N = 8	542±78.2 N = 9	920.1±126.9* N = 7
**NxD**	72.2±5.5 N = 6	437±71.7^$^ N = 5	577±112.3 N = 6	1600.8±302.3*^$^^#^ N = 6

Data is presented as mean±SD

* *p*<0.05 vs sham

$ *p*<0.05 vs Nx

# *p*<0.05 vs NxE (p<0.05). LV: left ventricle, EF: ejection fraction, EDV: end diastolic volume, SV: stroke volume.

### Significant correlations

A strong inverse correlation was found between AT_1_R PET kidney DV value and albuminuria (r = -0.731, *p* = 0.0009) also between AT_1_R PET DV kidney value and LV mass (r = -0.733, *p* = 0.0019). Interestingly, a weak negative correlation was found between the AT_1_R PET DV and SBP measurements (r = -0.28, *p* = 0.28).

## Discussion

In this study, we used 5/6 nephrectomy rat model to investigate AT_1_R alterations in CKD with PET. The major finding is the *in vivo* demonstration of renal AT_1_R reduction associated with the activated RAS due to CKD-induced hypertension in Nx rats, which was normalized following administration of the ACEI enalapril. RAS inhibition with ACEI was selected in this study instead of an ARB for management of CKD so as not to interfere with the binding of the losartan analog radiotracer used in PET imaging. The PET quantification was validated by *in vitro* ^125^I-[Sar^1^, Ile^8^]Ang II autoradiography studies.

Subtotal Nx rats developed hypertension, renal dysfunction and albuminuria at 18–20 weeks post-surgery. In addition to being an important prognostic factor in progressive CKD, albuminuria is considered as an independent predictor of cardiovascular morbidity and mortality [44, 45]. We have recently reported that induction of Nx in rats caused elevations in SBP and plasma Cr without evidence of albuminuria at 8–10 weeks post-surgery indicating intact glomerular barrier at that stage [31]. In the current work, development of significant albuminuria was observed with sustained hypertension and renal impairment at 18–20 weeks post-surgery. Consistent with previous clinical and experimental findings [46, 47, 33], the administration of ACEI enalapril normalized SBP, plasma Cr, and attenuated albuminuria associated with Nx.

The presence of heightened systemic and local RAS activity in CKD is well established [48, 36]. It can be postulated that maladaptive changes in both the circulatory and tissue RAS activity seem to be a driving force in the pathogenesis of CKD. In this work, the low levels of kidney Ang II can be attributed to the assessment of Ang II in homogenized total kidney tissue, which does not allow assessment of the concentrations within specific compartments. Thus, an increase in Ang II in some areas of the remnant kidney may not be detected if levels are decreased in other locations [49, 50].

Chronic administration of enalapril resulted in decreasing Ang II in the kidney while unexpectedly Ang II plasma levels were not changed. Normally the kidneys have much greater tissue concentrations of Ang II that is mainly produced through local ACE-dependent pathways. Therefore the administered dose of enalapril has been sufficient to suppress the conversion of Ang I to Ang II in the kidney only whereas displayed minimal effect on other organs which may have attenuated the reduction of plasma Ang II [49]. Another possible theory could be related to a feedback mechanism process compensating for the inhibition of ACE mediated synthesis of Ang II, either by decreasing the degradation of Ang II or by increasing alternative pathways of Ang II synthesis [51, 52]. Nevertheless, the effect of enalapril was sufficient to increase AT_1_R densities to normal levels in the remnant kidney by attenuating processes of Ang II-induced AT_1_R downregulation, confirming an inverse correlation between Ang II levels and AT_1_R expression.

Previous experimental studies investigating the renal RAS (mainly AT_1_R expression) in various animal models of CKD have yielded conflicting results that may reflect differences in the methodology and/or the time points of measurements [36, 53, 37]. Joly et al. reported a decrease (30–40%) in AT_1_R mRNA levels in cortex and outer medulla at 4 weeks post-surgery [53]; however, another study found no change in AT_1_R protein expression at the same time point [36]. Whereas, an increase (>70%) in AT_1_Rs was obtained using Western blotting at 8 weeks post-surgery by Kujal et al (2010) [37]. A non-invasive molecular imaging probe will provide relevant information regarding the dynamic changes in tissue AT_1_R levels.

In prior work, we have shown reduced [^18^F]FPyr-losartan retention (-23%) in the hypertrophied remnant kidney of Nx rats at 8–10 weeks compared to shams [31]. In this study, the -28% reduction obtained at 18–20 weeks with the same tracer was comparable to the early timepoint, indicating sustained reduced expression of renal AT_1_Rs in the untreated Nx rat. These reduced PET renal AT_1_R levels in Nx were normalized by enalapril, perhaps due to effective ACE inhibition that was sufficient to attenuate Ang II-induced AT_1_R downregulation. On the other hand, the deleterious effect of diltiazem on the plasma and kidney components of Ang II was associated with persistent reduction of AT_1_R kidney expression (similar to Nx rats) which could also provide an explanation for worsening of renal and cardiac functions in this group. PET imaging findings were supported by *in vitro* autoradiographic binding studies; thus, demonstrating reliable and accurate quantification of the dynamic changes of renal AT_1_Rs. This outcome emphasizes the benefits of using small animal PET in experimental work as it allows the usage of the same animal for longitudinal *in vivo* assessment of AT_1_R in health and disease states, and monitor effect of therapy.

The renal blood flow was reduced displaying a similar trend to [^18^F]FPyr-losartan decreased retention in the untreated Nx rats when compared with shams. However, this reduction in blood flow is not assumed to be the cause for diminished [^18^F]FPyr-losartan uptake, since the AT_1_R reduction with PET was confirmed by [^125^I]-[Sar^1^, Ile^8^]Ang II autoradiography. This assumption is further validated by the fact that using the same model of CKD, Nx rats exhibited no change in the renal blood flow associated with the PET AT_1_R reduction at 8–10 weeks post-surgery [31]. Indeed, local factors as Ang II, tumor growth factor-beta (TGF-βa) [24] and endothelin-1 [54], or reduction of nitric oxide production [55] among others, can be implicated in the changes in the renal hemodynamics.

Cardiac hypertrophy is a typical complication of CKD in response to the persistent hypertension and activation of the systemic RAS [8, 56]. Compared to shams, untreated Nx rats displayed significant increase in LV mass and diminished EDV with consequent decrease in SV presumably as a result of reduction of ventricular compliance due to the developed concentric hypertrophy [56]. The Ang II levels were not increased in the LV, hence the cardiac hypertrophy cannot be attributed to local activation of RAS in this study. However, other research groups suggested a role for cardiac RAS in LV hypertrophy associated with CKD [57, 7]. Nonetheless, systemic factors like anemia, dyslipidemia, and endothelial dysfunction or activated hormonal pathways such as the sympathetic nervous system that are known to be associated with CKD patients [58, 59] may have contributed to the promoting of LV hypertrophy [56, 60]. This result may also indicate the establishment of diastolic dysfunction [58], however such possibility was not further analyzed with echocardiography in this work. Interestingly, treatment with enalapril had no effect on LV levels of Ang II, however it prevented the progression of cardiac hypertrophy and partially improved cardiac efficiency.

The use of non-dihydropyridine CCB diltiazem was equally effective as antihypertensive therapy but failed to correct plasma Cr or albuminuria, and further increased their levels compared to untreated Nx rats. PET and autoradiographic renal AT_1_R expression results were not normalized in the diltiazem group, due to persistent elevation in renal Ang II levels. Moreover, diltiazem significantly increased Ang II levels in plasma and heart, although the mechanisms were not investigated. Cardiac hypertrophy was exacerbated in NxD rats compared to untreated Nx. However, SV was improved possibly due to normalization of SBP by diltiazem, despite high levels of Ang II in the heart. Consistent with previous research [61, 62, 34, 63], we demonstrated that late treatment with CCBs may further impair renal function and proteinuria in CKD. An interpretation for this effect can be due to their potent vasodilatory effect on the afferent arteriole dependent on pressure-induced depolarization and calcium entry through voltage-gated calcium channels [64, 65] which can have additive harmful effect induced by reduced renal mass [66, 62], and completely abolish renal autoregulatory capacity in the remnant kidneys of Nx rats [67, 62].

In summary, the Nx rat model of CKD exhibited renal impairment, proteinuria, sustained hypertension and cardiac hypertrophy. This was associated with elevated Ang II levels in kidney and compensatory downregulation of renal AT_1_Rs as measured *in vivo* by PET imaging and *in vitro* autoradiography. As expected, delayed administration of ACEI enalapril attenuated renal impairment, hypertension and prevented progression of cardiac hypertrophy. This was successfully accomplished through reduction of kidney Ang II levels and consequent normalization of renal AT_1_R. On the other hand, use of the non-dihydropyridine CCB diltiazem was equally effective in reducing SBP but did not normalize renal AT_1_R expression. Diltiazem induced increases in Ang II levels in plasma, kidney and heart, associated with exacerbation of renal and cardiac dysfunction. Hence, PET can provide insights about drug responses at AT_1_R level, which may help identifying patients likely to respond, thereby optimizing outcome and reducing adverse effects.

In conclusion, strong *in vivo* evidence is provided in this experimental study on the beneficial effects of ACEI enalapril in reducing Ang II combined with a normalization of renal AT_1_R levels and hypertension. Additionally, our data reinforces the need for early intervention to prevent progression of CKD and subsequent reduction in the risk for cardiovascular morbidities and mortalities. This outcome adds value to the feasibility of non-invasive [^18^F]FPyr-losartan PET for determination of receptor abnormalities with progression of the disease and monitoring of therapy.
